# CXCR4 expression in papillary thyroid carcinoma: induction by nitric oxide and correlation with lymph node metastasis

**DOI:** 10.1186/1471-2407-8-274

**Published:** 2008-09-30

**Authors:** Hironao Yasuoka, Rieko Kodama, Mitsuyoshi Hirokawa, Yuuki Takamura, Akira Miyauchi, Tokio Sanke, Yasushi Nakamura

**Affiliations:** 1 Department of Clinical Laboratory Medicine, Wakayama Medical University, Wakayama, Japan; 2 Department of Pathology, Kuma Hospital, Kobe, Japan; 3 Department of Surgery, Kuma Hospital, Kobe, Japan

## Abstract

**Background:**

Metastasis to regional lymph nodes is a common step in the progression of cancer. Recent evidence suggests that tumor production of CXCR4 promotes lymph node metastasis. Nitric oxide (NO) may also increase metastatic ability in human cancers.

**Methods:**

Nitrite/nitrate levels and functional CXCR4 expression were assessed in K1 and B-CPAP papillary thyroid carcinoma (PTC) cells after induction and/or inhibition of NO synthesis. CXCR4 expression was also analyzed in primary human PTC. The relationship between nitrotyrosine levels, which are a biomarker for peroxynitrate formation from NO in vivo, CXCR4 expression, and lymph node status was also analyzed.

**Results:**

Production of nitrite/nitrate and functional CXCR4 expression in both cell lines was increased by treatment with the NO donor DETA NONOate. The NOS inhibitor L-NAME eliminated this increase. Positive CXCR4 immunostaining was observed in 60.7% (34/56) of PTCs. CXCR4 expression was significantly correlated with nitrotyrosine levels and lymph node metastasis in human PTC.

**Conclusion:**

Our data indicate that NO stimulates CXCR4 expression in vitro. Formation of the NO biomarker nitrotyrosine was also correlated with CXCR4 expression and lymph node metastasis in human PTC. NO may induce lymph node metastasis via CXCR4 induction in papillary thyroid carcinoma.

## Background

Nitric oxide (NO) is a pleiotropic regulator and inflammatory stimulant, critical to numerous biological processes, including vasodilatation, neurotransmission, and macrophage-mediated immunity [1]. It also has both genotoxic and metastasis-promotng properties. Increased NO generation in cancer cells may contribute to tumor hemangiogenesis or lymphangiogenesis by up-regulating vascular endothelial growth factor (VEGF) [2], VEGF-C [3], or VEGF-D [4]. The effects of NO are mediated in part by its metabolites, such as peroxynitrite. Peroxynitrite can oxidize and nitrate DNA and can also nitrate tyrosine in proteins to produce nitrotyrosine [5]. Thus the presence of nitrotyrosine in tissues has been used as a biomarker for peroxynitrite formation in vivo from NO.

Metastasis to regional lymph nodes is a common step in the progression of cancer. Metastasis of cancer cells is a complex process involving invasion, hemangiogenesis, lymphangiogenesis, trafficking of cancer cells through blood or lymph vessels, extravasations, organ-specific homing, and growth. Recent evidence suggests that metastatic cancer cells overexpress CXC chemokine receptor 4 (CXCR4), and that CXCR4 plays a critical role in homing of cancer cells to specific metastatic sites[6]. The CXCR4 ligand CXCL12 was found to be expressed in liver, bone marrow, lung, and lymph nodes. Furthermore, metastasis of cancer cells to regional lymph nodes and lung in immunodeficient mice were inhibited by a neutralizing antibody against CXCR4 [6]. However, how CXCR4 expression is regulated is largely unknown. We considered the possibility that the inflammatory stimulant NO is involved in the expression of CXCR4 in papillary thyroid carcinoma (PTC) because NO has been shown to up-regulate the expression of prometastatic and proangiogenic genes including VEGF [2], VEGF-C [3], and VEGF-D [4]. In experimental tumor models, a contributory role of NO in tumor metastasis has been also demonstrated [7]. In addition, signal-activated transcription factor NF-kappa B, which is linked to NO signaling pathways, has been shown to up-regulate the expression of CXCR4 and to mediate CXCL12-induced T cell migration [8,9]. Furthermore approximately one third of PTCs show moderate to marked lymphocytic infiltration. In most instances, this probably represents a host reaction to the tumor; in others, it may be due to a preexisting autoimmune thyroiditis [10]. The presence of NO in tumor cells or the tumor micro-environment may increase the metastatic ability of PTC, in which many cases have inflammatory infiltration.

In this study, incubation of K1 and B-CPAP PTC cells with an NO donor resulted in induction of functional CXCR4 expression. This induction was significantly inhibited by addition of the NOS inhibitor L-NAME. In addition, we investigated how CXCR4 expression relates to lymph node metastasis and nitrotyrosine formation in PTC.

## Methods

### Cell culture

The K1 and B-CPAP PTC cell lines were purchased from the European Collection of Cell Cultures (ECACC, Wiltshire, United Kingdom) and the German Collection of Microorganisms and Cell Cultures (DSMZ, Braunschweig, Germany), respectively. K1 cells were maintained at 37°C in 5% CO_2_, as monolayers in tissue culture dishes containing DMEM: Ham's F12 (Invitrogen, Tokyo, Japan) supplemented with 10% heat-inactivated fetal calf serum (FCS) (HyClone, South Logan, UT, USA) as described previously [4]. B-CPAP cells were maintained at 37°C in 5% CO_2_, as monolayers in tissue culture dishes containing DMEM (Invitrogen) supplemented with 10% heat-inactivated FCS (HyClone). For the experiments, 6 cm tissue culture plates (Corning, Corning, NY, USA) were seeded with 3 × 10^5 ^cells in 3 ml of medium + 10% FCS. Medium was changed (day 3), and when the cells were subconfluent (day 5), 5 mM (B-CPAP) or 10 mM (K1) L-NAME (Sigma-Aldrich, Tokyo, Japan), if administered, was added 2 h before 1 mM DETA NONOate (Cayman Chemical, Ann Arbor, MI, USA). These concentrations of L-NAME or DETA NONOate had no effect on cell viability as measured by the CellTiter 96 Aqueous One Solution Cell Proliferation Assay (Promega, Madison, WI, USA) (data not shown).

### Measurement of nitrate/nitrite production

Measurement of nitrate/nitrite production was performed as described previously [4]. After DETA NONOate administration, cells were incubated for 4, 6, 8, 12, 16, and 24 h. The supernatants were collected and centrifuged to remove cell debris. The amount of nitrate/nitrite was determined using **a **Nitrate/Nitrite Fluorometric Assay Kit (Cayman Chemical) [11].

### Determination of CXCR4 mRNA production

After DETA NONOate administration, cells were incubated for 4, 6, 8, 12, 16, and 24 h. Total RNA was extracted using Trizol (Invitrogen) according to the protocol provided by the manufacturer. After DNase treatment using DNA-free™ (Ambion, Austin, TX, USA), mRNA was reverse-transcribed for single strand cDNA using Oligo-(dT)_20 _primer and Thermoscript (Invitrogen, Tokyo, Japan) as described previously[4]. CXCR4 transcription was measured by quantitative real-time PCR of the resulting cDNA, using universal TaqMan PCR reagents, and an ABI Prism 7000 sequence detector equipped with a 96-well thermal cycler (Perkin-Elmer Applied Biosystems, Foster City, CA, USA). The primer and probe mixtures for CXCR4 and GAPDH were purchased from Perkin-Elmer Applied Biosystems, and PCR was carried out according to the manufacturer's protocol. CXCR4 mRNA expression was quantitated relative to control cells (treated with neither DETA NONOate or L-NAME) based on a real-time PCR standard curve constructed with serially diluted solutions of a CXCR4 cDNA-containing plasmid as templates. All experiments were performed in triplicate, although amplification results have been shown to be very consistent and tube-to-tube-variability very low. Mean values were used for statistical testing. The GAPDH transcript levels of each sample were also monitored, which confirmed that tube-to-tube-variability was very low (data not shown).

### Determination of CXCR4 protein production

For the determination of CXCR4 protein production, cells were incubated for 8 h (K1) or 12 h (B-CPAP) after DETA NONOate administration, and harvested as described above. Cell lysates were prepared using T-PER™ Tissue Protein Extraction Kit (Pierce, Rockford, IL, USA) containing Halt™ Protease Inhibitor Cocktail (Pierce) as described previously [4]. For Western blot analysis of CXCR4, 30 microgram samples of whole cell lysate were separated by electrophoresis on 10–20% SDS polyacrylamide gels and transferred to PVDF membrane by electroblotting. The membrane was blocked with 5% skim milk in PBS for 1 h at room temperature, incubated overnight with anti-human CXCR4 rabbit antibody (Abcam Ltd., Cambridge, UK), rinsed with PBS, and labeled with peroxidase-conjugated anti-rabbit secondary antibosdy (Dako Cytomation, Denmark) for 1 h at room temperature. The signals were visualized using the LumiGLO Reserve chemiluminescence substrate kit (KPL, Inc, Gaithersburg, MD, USA) and recorded by luminocapture (ATTO, Tokyo, Japan). Anti-beta-2-microglobulin antibody (Dako Cytomation) was used for the internal control. To compare levels of proteins, the density of each band was measured by densitometry.

### Chemoinvasion assay

Chemoinvasion assay of each cell type was assessed in BD Matrigel™ Invasion Chamber 24-well plates (BD Biosciences, Bedford, MA, USA). Briefly, the upper compartment of the chamber was seeded with 2 × 10^4 ^cells, and the lower compartment of the chamber was loaded with medium. Medium in the upper compartment of the chamber was changed (day 3), and 5 mM (B-CPAP) or 10 mM (K1) L-NAME, if administered, was added 2 h before 1 mM DETA NONOate. At the same time, the lower compartment of the chamber was loaded with different concentrations of recombinant human CXCL12 (rhCXCL12) or without rhCXCL12 (R&D Systems, Minneapolis, MN, USA). The two compartments were separated by a Matrigel-coated membrane (10 micrometer thickness and 8 micrometer pore size). Uncoated membranes were used as a control for non-invasive cell migration, in accordance with the manufacturer's directions. After the incubation (day5), the chamber was removed, and cells that had migrated to the bottom of the membrane were fixed and stained in Cyto Quick Solution (Muto Pure Chemical, Tokyo, Japan) and counted by light microscopy. The percentage of invading cells after incubation (% Invasion) was calculated as (Matrigel)/(Control membrane), according to the manufacturer's protocol.

### Patients and tumor samples

After investigational review board approval by the Wakayama Medical University Medical Ethics Committee and informed consent was obtained, paraffin-embedded archival specimens from 56 patients with PTC, who were diagnosed and treated in Kuma Hospital, Japan in 2003, were examined immunohistochemically. None of these cases had a family history of thyroid cancer or malignancy. The patients had received total or subtotal thyroidectomy with regional lymphadenectomy (central neck dissection, lateral neck dissection, superior mediastinal dissection, or a combination of the above). All sections of the excised tumors were histologically evaluated by 3 pathologists (HY, RK, and YN). Of the 56 patients selected, 49 had classical PTC, 3 had a poorly differentiated form, and 4 had a follicular variant as assessed by histopathology. Patients with multifocality were excluded. Patients and tumor characteristics are shown in Table 1. The median age at surgery for the selected patients was 49.5 years (range, 16–76 years). All of the patients have been followed-up and none has had a recurrence.

**Table 1:** Clinico-pathological data for 56 papillary thyroid carcinoma cases and relationship between CXCR4 expression and covariates

Factors		CXCR4 expression		*p *value
		Negative	Positive	

Age	<45	10	11	0.4009
	≧45	12	23	
Sex	Male	4	3	0.4149
	Female	18	31	
Histology	Well Diff.	20	29	0.1989
	Follicular variant	2	2	
	Poorly Diff.	0	3	
Tumor size	pT1	1	6	0.1585
	pT2	16	21	
	pT3	3	1	
	pT4	2	6	
Lymph node Metastasis	pN0	10	4	0.0116
	pN1a	3	12	
	pN1b	9	18	
Nitrotyrosine Levels	Low grade	19	8	<0.001
	High grade	3	26	

*Tumor size, lymph node metastasis, and distant metastasis were classified according to the TNM classification of the UICC, 2002

*Fisher's exact test was used to examine the association between CXCR4 expression and covariates.

*Diff.: Differentiated

### Immunohistochemistry

For immunostaining, paraffin sections of 4-micrometer thickness were de-paraffinized, placed in a solution of 97% methanol and 3% hydrogen peroxide for 5 min, then autoclaved for antigen retrieval. After washing in PBS, the slides were treated for 20 min with Protein Block Serum-free (DAKO Cytomation, Carpinteria, CA, USA). This was followed by an overnight incubation at 4°C. in a humidified chamber with a 1:30 diluted anti-human CXCR4 rabbit antibody (Abcam). After the overnight treatment, to avoid the nonspecific biotin reaction, we used Histofine Simple Stain MAX PO (NICHIREI, Tokyo, Japan) as the second antibody for 60 min according to the manufacturer's instructions. Color was developed using diaminobenzidine with 0.01% hydrogen peroxide. Hematoxylin was used as a counterstain. For the negative control, all reagents except for the primary antibody were used.

The immunohistochemical scoring was performed blindly by 3 pathologists (HY, RK, and YN) who had no clinical knowledge of the patients. The immunostained sections were scanned by light-microscopy. Cytoplasmic labeling of tumor cells was classified as either negative (if no staining or positive staining was present in <10% of tumor cells) or positive (if over 10% of tumor cells stained positively).

### Statistics

The effects of drug treatment were analyzed by ANOVA followed by Student's t test. Fisher's exact test was used to examine the association between CXCR4 immunoreactivity and other clinicopathological factors. A p value less than 0.05 was considered significant. A software package (JMP IN 5.1.1, SAS Institute, Cary, NC, USA) was used for all statistical testing and management of the database.

## Results

### Effects of NO on CXCR4 expression

**Figure 1:**
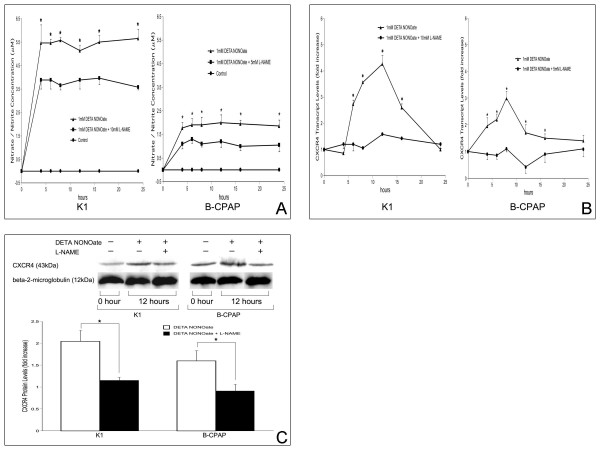
**Effects of NO on CXCR4 expression**. Effects of DETA NONOate in the presence or absence of L-NAME on (A) nitrate/nitrite production, (B) CXCR4 mRNA expression, and (C) CXCR4 protein expression. K1 and B-CPAP cells were treated with 1 mM DETA NONOate in the presence or absence of L-NAME for various time periods and prepared for (A) measurement of nitrate/nitrite production, (B) real-time RT-PCR analysis, and (C) western blot analysis, as described in Materials and Methods. Determinations were performed in triplicate and expressed as mean of three experiments ± SE. (A) Control indicates cells with no treatment. (B-C) Data was expressed as ratio of mRNA or protein levels relative to control (untreated) cells. * indicates significant difference (p < 0.05) from control and/or L-NAME-treated cells.

To examine the effect of NO on CXCR4 induction, K1 and B-CPAP cells were treated with the NO donor DETA NONOate. A significant increase in nitrate/nitrite production in the supernatants after stimulation with DETA NONOate was observed (Figure 1A). Pre-treatment of the cells with the NOS inhibitor L-NAME significantly inhibited this increase. As shown in Figures 1B and 1C, a significant increase in CXCR4 mRNA and protein expression was observed after stimulation with DETA NONOate. Pretreatment with L-NAME substantially inhibited the effects of DETA NONOate on CXCR4 mRNA and protein expression.

### NO regulates CXCL12-mediated chemoinvasion

**Figure 2:**
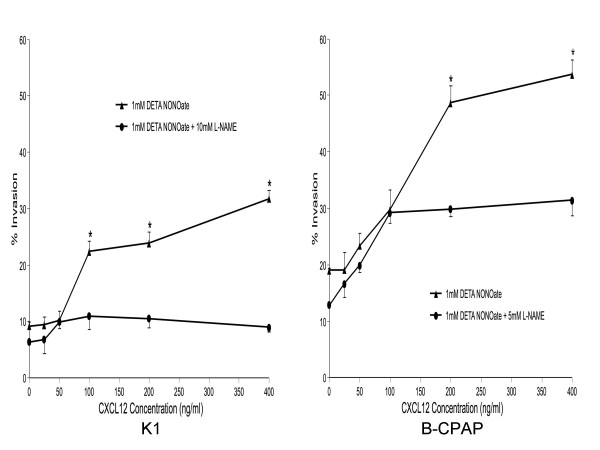
**CXCL12 induced the chemoinvasion of papillary thyroid cancer cells**. Cells were seeded into the upper compartments of Matrigel Invasion Chambers as described in Materials and Methods. Uncoated or Matrigel-coated membranes separated the upper compartments from lower compartments containing the indicated concentrations of CXCL12. Cells were treated with DETA NONOate with or without L-NAME. Two days later, cells that had migrated to the bottom of the membrane were stained and counted. The percentage of invasive cells (% Invasion) was calculated as the number of cells penetrating the Matrigel-coated membranes divided by the number penetrating the uncoated membranes. Determinations were performed in triplicate and expressed as mean of three experiments ± SE. * indicates significant difference (p < 0.05) from L-NAME-treated cells.

To determine the role of NO in cellular response to the CXCR4 ligand CXCL12, we examined the effect of L-NAME on rhCXCL12- and DETA NONOate-induced in-vitro Matrigel invasion by K1 and B-CPAP cells (Figure 2). rhCXCL12 produced a dose-dependent increase in invasiveness, which was inhibited in cells pretreated with L-NAME.

### CXCR4 expression in papillary thyroid carcinoma tissue

**Figure 3:**
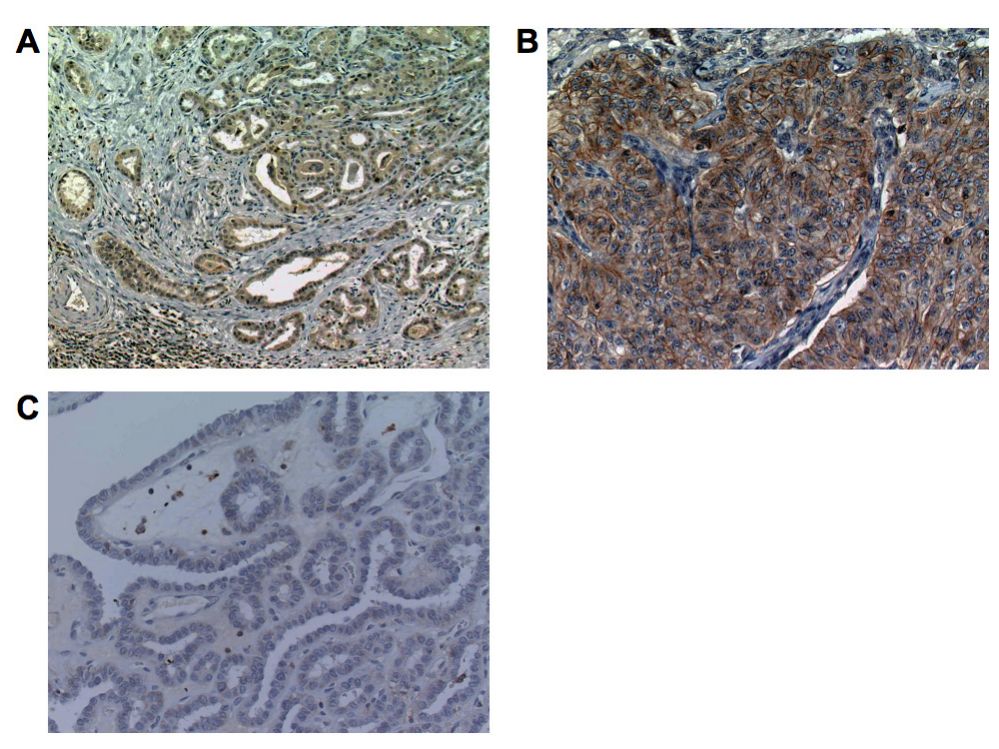
**CXCR4 expression in human papillary thyroid carcinoma tissue**. (A) immunohistochemical localization of CXCR4 protein was cytoplasmic. (B) In some cases carcinoma cells showed positive staining in the membrane. (C) In the tumor in which CXCR4 immunodetection are unreactive, staining for CXCR4 occurred in inflammatory cells.

In this study, immunohistochemical localization of CXCR4 protein was cytoplasmic (Figure 3A). In these cases, tumor CXCR4 expression was homogenous. In some cases carcinoma cells showed positive staining in the membrane in addition to cytoplasmic staining (Figure 3B). According to the criteria for CXCR4 immunohistochemical evaluation, CXCR4 positive staining was detected in 34 cases (60.7%). In the tumor in which CXCR4 immunodetection are unreactive, staining for CXCR4 occurred in inflammatory cells (Figure 3C). In contrast, very little or no staining was observed in normal thyroid epithelium (data not shown).

### CXCR4 expression is correlated with nitrotyrosine levels and lymph node metastasis

We have previously reported that nitrotyrosine formation was detected by immunohistochemistry in all PTCs [4]. The intensity of nitrotyrosine immunostaining was evaluated by dividing the cytoplasmic staining reaction into four groups: 1 = weak; 2 = moderate; 3 = strong; and 4 = very strong. The fraction of immunostainined cells was evaluated as follows: 1 = <25%; 2 = 25–50%; 3 = 50–75%; and 4 = >75% of tumor cells showing cytoplasmic staining. These summed scores were then divided into two groups as low-grade (2–4) and high-grade (5–8) for statistical testing. High-grade nitrotyrosine staining was observed in 51.8% (29/56) of the PTCs, and its expression was significantly correlated with lymph node metastasis (p = 0.0173), as shown in Table 1. CXCR4 expression was correlated with high-grade nitrotyrosine staining (p < 0.001) and lymph node metastasis (p = 0.0116). There was no significant correlation between CXCR4 expression and other clinicopathological factors.

## Discussion

NO, an inorganic free radical gas, is an important signaling molecule and bioactive agent that mediates vasodilatation and various other actions such as neurotransmission and host defense [1]. NO produced by macrophages can have antibacterial and antitumor functions, however, chronic induction of NO and NO synthase may contribute to many pathological processes including inflammation and cancer [12,13]. In experimental tumor models, a contributory role of NO in tumor metastasis has been also demonstrated [2,7]. Previous reports have shown that NO induces lymphangiogenic factor VEGF-C or VEGF-D expression in vitro and in vivo, and may play an important role in lymph node metastasis in cancer [3,4,14]. VEGF-D in particular may have an important role in particular for lymph node metastasis in PTCs [15]. CXCR4 is the physiological receptor for CXCL12, which belongs to a chemokine family that has potent chemotactic activity for lymphocytes. It is well known that peripheral lymphocytes preferentially localize to peripheral lymphoid tissues, such as lymph nodes; this is called the homing phenomenon [16]. Recent evidence suggests that metastatic cancer cells overexpress CXCR4 and that this receptor plays a critical role in homing of cancer cells to specific metastatic sites [6].

In this study, the NO donor DETA NONOate induced CXCR4 mRNA and protein expression in the K1 and B-CPAP PTC cell lines. In chemoinvasion assays, rhCXCL12-induced invasion was observed in both cell lines after treatment with DETA NONOate, indicating that DETA NONOate stimulated functional CXCR4 expression. All these increases were significantly inhibited in the presence of the NOS inhibitor L-NAME. A significant increase in nitrate/nitrite production in the supernatants after stimulation with DETA NONOate was also observed, and treatment of cells with L-NAME substantially inhibited this increase as well. Our results suggest that CXCR4 expression may be regulated by NO in these PTC cell lines.

In addition, positive CXCR4 immunostaining was observed in 60.7% (34/56) of PTCs. An earlier report of CXCR4 expression in cultured cell lines obtained originally from PTC also supports our present observation [17]. In the present study, immunohistochemistry revealed that CXCR4 expression was significantly correlated with nitrotyrosine levels and lymph node metastasis in human PTC tumor samples. Our findings suggest that CXCR4 expression in human PTC may be associated with nitrotyrosine formation and lymph node metastasis. In this study, the NO donor DETA NONOate induced functional CXCR4 protein expression in the both K1 and B-CPAP PTC cell lines. These results may support the correlation between CXCR4 expression and nitrotyrosine formation in human PTC tumor samples. Consistent with up-regulation of CXCR4 expression by NO, 70% of the low nitrotyrosine patients showed negative CXCR4 staining. However, 10% of the high nitrotyrosine patients also showed negative cytoplasmic staining. Although this is lower than the overall percentage showing negative cytoplasmic staining (48%), it indicates that other factors regulate CXCR4 expression in these patients. As it has been reported previously that CXCR4 expression is induced by other factors, such as NF-kappa B [8], c-erbB-2 [18], or hypoxia-inducible factor 1 [19], these factors may induce CXCR4 expression in the cases with low levels of nitrotyrosine.

Recently Balabanian et al demonstrated that CXCL12 can also be a ligand for CXCR7 [20]. A previous report also showed that CXCR7 expression in tumor cells enhances cell growth, survival, and adhesion, and also causes tumor growth in vivo in animal models [21]. Since CXCL12 is expressed preferentially in lymph nodes [6], this may support our finding that CXCR4 expression was significantly correlated with lymph node metastasis in human PTC tumor samples. As CXCR7 expression promotes cancer metastasis in various human cancers [22,23], it would be also important to investigate the role of CXCR7 in PTC in the future study.

## Conclusion

Nitric oxide induces functional CXCR4 in vitro, and levels of the NO marker nitrotyrosine are correlated with CXCR4 expression and lymph node metastasis in human papillary thyroid carcinoma. NO may play an important role in metastasis of this cancer via CXCR4 induction.

## Abbreviations

CXCR: CXC chemokine receptor; PTC: Papillary Thyroid Carcinoma; VEGF: Vascular Endothelial Growth Factor; NO: Nitric Oxide; DMEM: Dulbecco's Modified Eagle Medium; FCS: Fetal Calf Serum; L-NAME: N^G^-nitro-L-arginine methyl ester; DETA NONOate: (Z)-1-[2-(2-Aminoethyl)-N-(2-ammonioethyl)amino]diazen-1-ium-1,2-diolate; NOS: NO synthases; PBS: phosphate-buffered saline; RT: reversetranscription; PCR: polymerase chain reaction; GAPDH: glyceraldehyde-3-phosphate dehydrogenase; rhCXCL12: recombinant human CXCL12.

## Competing interests

The authors declare that they have no competing interests.

## Authors' contributions

HY conceived of the study, participated in the design of the study, conducted and evaluated both the immunostainings and the in vitro assay, performed the statistical analysis and drafted the manuscript. RK contributed to the design of the study, evaluated the immunostainings, and helped to draft the manuscript. MH, YT, and AM participated in the design and coordination of the study. TS contributed to the design of the study and interpretation of the results. YN participated in the design, evaluated both the immunostainings and the in vitro assay, and helped to draft the manuscript. All authors read and approved the final manuscript.

## Pre-publication history

The pre-publication history for this paper can be accessed here:


